# Carbon ion radiotherapy for unresectable primary or recurrent soft tissue sarcomas: long-term outcomes based on the local effect model

**DOI:** 10.1186/s12885-026-16213-w

**Published:** 2026-06-04

**Authors:** Ping Li, Yongqiang Li, Cihang Bao, Xin Cai, Zheng Wang, Qing Zhang

**Affiliations:** 1 Department of Radiation Oncology, Shanghai Proton and Heavy Ion Center, Shanghai, China; 2 Shanghai Engineering Research Center of Proton and Heavy Ion Radiation Therapy, Shanghai, China; 3 Shanghai Key Laboratory of Radiation Oncology (20dz2261000), Shanghai, China; 4 Department of Medical Physics, Shanghai Proton and Heavy Ion Center, Shanghai, China

**Keywords:** Carbon Ion Radiotherapy, Soft tissue sarcoma, Local Effect Model, Neutrophil-to-lymphocyte ratio

## Abstract

**Background:**

The application of carbon ion radiotherapy (CIRT) for unresectable primary or recurrent soft tissue sarcoma (STS) remains controversial and existing data are limited. We herein present our institutional experience with CIRT for unresectable primary or recurrent STS.

**Methods:**

We retrospectively evaluated the outcomes of CIRT in unresectable primary or recurrent STS patients at our center. We assessed the 4—and 5—year local control (LC), overall survival (OS), distant metastasis free survival (DMFS), progression free survival (PFS), as well as acute and late toxicities. Additionally, we analyzed the prognostic factors associated with the treatment outcomes.

**Results:**

Between June 2015 and August 2022, 48 consecutive patients with unresectable primary (*n* = 8, 16.7%) or recurrent (*n* = 40, 83.3%) STS were treated with CIRT. The most common tumor sites were the retroperitoneum and pelvis (75.0%). The predominant histological subtypes included liposarcoma (20.8%), leiomyosarcoma (10.4%), synovial sarcoma (8.3%) and undifferentiated pleomorphic sarcoma (8.3%). Nine patients (18.8%) had a history of prior in-field radiotherapy. The median follow-up duration was 48.6 months. The 4-year LC, OS, DMFS, and PFS rates were 65.8%, 59.7%, 50.3%, and 36.7%, respectively. The 5-year LC, OS, DMFS, and PFS rates were 60.3%, 54.7%, 44.0%, and 28.0%, respectively. The median survival time was 67.3 months. Late toxicities included grade 3 dermatitis (*n* = 2, 4.2%), grade 3 decreased joint range of motion (*n* = 1, 2.1%), and grade 3 peripheral neuropathy (*n* = 1, 2.1%). Patients who received a higher prescribed dose (biologically effective dose, BED ≥ 129 Gy) exhibited significantly better LC (*p* = 0.010) and PFS (*p* = 0.004) compared to those who received a lower prescribed dose. A pre-CIRT neutrophil-to-lymphocyte ratio (NLR) ≥ 3 was significantly associated with inferior DMFS (*p* = 0.020).

**Conclusions:**

For select patients with unresectable primary or recurrent STS, CIRT represents a potentially effective and well-tolerated treatment option, though it requires careful evaluation of neural risks—particularly for lesions adjacent to critical nerve structures.

**Supplementary Information:**

The online version contains supplementary material available at 10.1186/s12885-026-16213-w.

## Introduction

Soft tissue sarcomas (STS) are a relatively uncommon and highly heterogeneous group of malignancies, accounting for approximately 1% of cancers diagnosed in adults [1]. These tumors are predominantly located in the extremities, retroperitoneum, and trunk. Complete surgical resection remains the standard treatment for patients with localized STS; however, achieving complete resection is often challenging or infeasible due to large tumor size or involvement of adjacent vital structures, particularly in retroperitoneal sarcoma (RPS) [2]. The management of patients with STS who are unsuitable for surgery or who experience recurrence after surgery is challenging. Currently, no standard treatment exists for these types of STS.

Radiotherapy may serve as a local treatment option for unresectable primary or recurrent STS; however, STS are considered to be a group of malignancies that are highly resistant to conventional radiotherapy. Carbon ion radiotherapy (CIRT) is a specialized treatment that offers unique physical and biological advantages over conventional radiotherapy [3]. Carbon ions exhibit a higher linear energy transfer (LET), leading to greater relative biological effectiveness (RBE) and more potent tumor cell killing. Furthermore, the Bragg peak phenomenon enables precise dose distribution, thereby minimizing damage to surrounding critical organs. These attributes render CIRT particularly effective for treating radio-resistant tumors and those located in close proximity to critical structures [4].

Despite its potential, the application of CIRT in STS, particularly in unresectable cases, remains limited. Imai et al. demonstrated that CIRT provides effective tumor control in unresectable STS, reporting a 5-year local control (LC) rate of 65% [5]. However, the CIRT in their study was based on the Microdosimetric Kinetic Model (MKM), which is primarily applied in Japanese facilities. In contrast, European centers and our institution primarily employ the Local Effect Model (LEM). These models differ substantially in their theoretical foundations, biological endpoints, and clinical parameters. The MKM is based on microdosimetric concepts and specific energy deposition, yielding an RBE that is largely independent of dose per fraction within the clinical range. In contrast, the LEM links biological effect to locally deposited dose, resulting in a pronounced dose-dependent RBE. Additional distinctions include the reference radiation, cell lines, and α/β ratios employed, which lead to significant differences in RBE-weighted dose distributions—especially at the distal edge and in high-gradient regions [6]. Emerging research indicates substantial differences in clinical outcomes between these two models [7, 8]. The optimal treatment dose and corresponding clinical outcomes for STS under the LEM model remain to be fully elucidated.

This retrospective study aims to evaluate the efficacy and safety of CIRT in patients with unresectable STS based on the LEM model. Furthermore, we investigated prognostic factors influencing outcomes following CIRT for STS, with a specific focus on the neutrophil-to-lymphocyte ratio (NLR). As a well-established biomarker of tumor-associated inflammation, NLR has been shown to correlate with prognosis in numerous malignancies [9]. However, its prognostic significance within the specific context of CIRT for STS has not yet been conclusively established.

## Patients and methods

### Study population

A consecutive cohort of patients with histologically confirmed unresectable primary or recurrent STS undergoing CIRT at the Shanghai Proton and Heavy Ion Center (SPHIC) between June 2015 and August 2022 was retrospectively analyzed. Inclusion criteria comprised patients aged 16 years or older with histologically confirmed unresectable primary or recurrent STS undergoing definitive CIRT. Exclusion criteria included: 1) lymph node metastasis; 2) distant metastasis. Each patient underwent complete blood counts (CBC) testing before and after CIRT. Pre-CIRT blood samples were collected immediately before the delivery of the first treatment fraction, and post-CIRT samples were obtained right after the completion of the final radiotherapy fraction. This study was approved by the Ethics Committee of Shanghai Proton and Heavy Ion Center (No. 260415EXP-01). Informed consent was obtained from all individual participants included in the study.

### Carbon ion radiotherapy

CIRT was administered with definitive intent. A simulation computed tomography (CT) scan with 2 mm slice thickness was performed, with patients positioned either supine or prone based on the tumor's anatomical location. The gross tumor volume (GTV) was delineated on the simulation CT or, when available, on T2-weighted magnetic resonance imaging (MRI) fused with the simulation CT. A clinical target volume (CTV) margin of 5–30 mm was then added, with adjustments made to protect critical structures including the bowel, bones, bladder, and rectum. The CTV was prescribed to receive at least 95% of the prescribed dose. The planning target volume (PTV) included the CTV plus a 3–5 mm margin.

CIRT was administered once daily for 5 days per week. The prescribed doses were based on the RBE-weighted dose (D_RBE_). Each plan was optimized in Syngo (V13C, Siemens, German) treatment planning system (TPS), and evaluated by a local effect model (LEM I) (Krämer and Scholz 2000) in the carbon-ion RT plans The total radiation dose ranged from 45 to 80.8 Gy, with a median of 70.4 Gy. Dose per fraction ranged from 3 to 4.6 Gy, with a median of 4 Gy. Thirteen patients received a total dose of 72 Gy delivered in 18 fractions. The detailed treatment protocol is provided in Supplementary Table S1. The D_RBE_ was determined by the treating radiation oncologist. The decision considered several factors: tumor volume and location, proximity to and tolerance of adjacent OARs, and history of prior radiotherapy. The primary goal was to deliver the maximum dose deemed safe to the target volume while respecting stringent constraints for OARs. The dose constraints for OARs were applied: maximum point dose to the bowel ≤ 55 Gy; for rectum, Dmax ≤ 105% of the prescription dose, D3cc < 60 Gy, D7cc < 55 Gy, and D10cc < 50 Gy. The Dmax to the sacral nerves was generally constrained to < 70 Gy where feasible.

To compare the biological dose intensity across different fractionation schemes, the biologically effective dose (BED) was calculated using the Linear-Quadratic (LQ) model. An α/β = 5 was selected based on the radiobiology of STS and its common use in photon radiotherapy studies for sarcoma [10, 11].

### Follow up and evaluation

Following completion of CIRT, patients underwent follow-up assessments every three months for the first three years and every six months thereafter. Follow-up assessments included physical examinations, routine blood tests, and MRI or CT scans of the lesion area. An annual chest CT scan was recommended. PET/CT and other examinations were performed based on clinical evidence of metastasis, recurrence, or other concurrent conditions. Acute and late toxicities were classified according to the highest grade observed within three months (acute) and after three months (late) following the initiation of CIRT. All toxicities were graded according to National Cancer Institute Common Terminology Criteria for Adverse Events (CTCAE) version 5.0. The follow-up period was calculated from the initial date of CIRT. CBCs were obtained, with particular attention to the absolute neutrophil and lymphocyte counts. The NLR was calculated by dividing the absolute neutrophil count by the absolute lymphocyte count. LC was defined as the absence of tumor progression or recurrence within the radiotherapy field. Progression free survival (PFS) was determined by the absence of local or distant disease progression. Distant metastasis free survival (DMFS) was defined as the absence of disease progression outside the radiotherapy field. Overall survival (OS) was defined as the time from the start of radiotherapy (RT) to death from any cause.

### Statistical analyses

Median follow-up was estimated using the reverse Kaplan–Meier method. LC, PFS, DMFS, and OS were estimated using the Kaplan–Meier method. The log-rank test was employed for univariate analysis. The following factors were evaluated: patient age, tumor location, history of previous radiotherapy, metal implants, radiation dose, tumor volume, and NLR before and after treatment. For univariate survival analysis, continuous variables (e.g., Age, BED, and GTV) were dichotomized at their median values. Given the diverse histology and limited case numbers for individual STS subtypes, no formal histology-stratified analysis was performed to avoid statistically underpowered comparisons. After consulting with statisticians, we omitted multivariate analysis in this study owing to the limited number of cases and events. A p-value < 0.05 was considered statistically significant. All statistical analyses were performed using R version 4.4.2.

## Results

A cohort of 48 patients treated with CIRT between June 2015 and August 2022 was included in this study. The patients’ characteristics are summarized in Table 1. The median age of these patients was 53.5 years (range: 16–86 years). There were 30 males and 18 females. Of the 48 patients, 40 (83.3%) had recurrent tumor after resection, 8 (16.7%) with primary unresectable tumor. Of the 40 patients who had recurrence tumor after surgery, 22 (55.0%) underwent at least two resections. Most tumors were located in retroperitoneum and pelvis (75.0%). The histological subtype included liposarcoma (20.8%), leiomyosarcoma (10.4%), synovial sarcoma (8.3%), undifferentiated pleomorphic sarcoma (8.3%), malignant peripheral nerve sheath tumor (6.3%), myxofibrosarcoma (6.3%) and other histologies with fewer than 3 cases each (41.7%). FNCLCC (French Federation of Cancer Centers Sarcoma Group) grading information was available for only 12 of the 48 patients (25.0%) due to the retrospective nature of data collection and the inclusion of cases from external institutions. Detailed subtype-level data, including the distribution of liposarcoma subtypes, are provided in Supplementary Table S2. Among the 48 patients, 9 (18.8%) had previously received in-field radiotherapy, 5 (10.4%) patients had metal implants within the irradiation field.

**Table 1:** Patients’ characteristics

Characteristics	No. of patients
**Median age (range), y**	53.5 (16–86) y
**Male: female ratio**	30:18
Tumor type	
Primary tumor without resection	8
Recurrent tumor after resection	40
Tumor site	
Retroperitoneum	19
Pelvis	17
Extremities	10
Trunk	2
Tumor size (range), cm3	
~ 100	15
100–200	14
200–500	11
500–1000	5
1000~	3
Histology	
Liposarcoma	10
Leiomyosarcoma	5
Synovial sarcoma	4
UPS	4
MPNST	3
Myxofibrosarcoma	3
Others	19
Metal implant	
Yes	5
No	43
Re-irradiation	
Yes	9
No	39
Surgery Times	
0	8
1	18
2	15
≥ 3	7

*UPS* undifferentiated pleomorphic sarcoma, *MPNST* malignant peripheral nerve sheath tumor

**Fig. 1:**
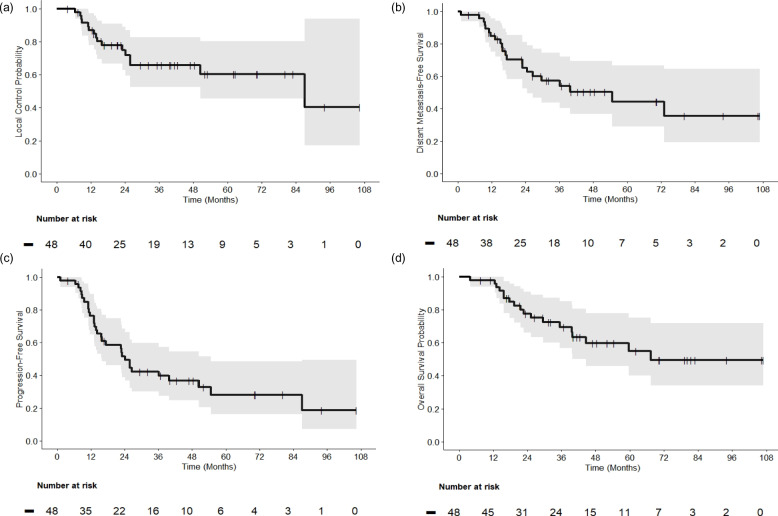
Kaplan–Meier analysis of local control (**a**), distant metastasis free survival (**b**), progression free survival (**c**), and overall survival (**d**) in 48 patients treated with carbon ion radiotherapy

The median follow up was 48.6 months. Eighteen patients (37.5%) had no evidence of local recurrence or distant metastases at the time of death or last follow-up. Across the entire cohort, 30 patients experienced disease progression, 8 patients developed local recurrence, 14 patients had distant metastases, and 8 patients exhibited both local and distant relapses. The median time to local recurrence was 23.8 months (range: 6.6–87.0 months) among the 16 patients. Eleven of the 16 local recurrences (68.8%) occurred within 2 years after CIRT. The 4- and 5-year LC rates were 65.8% (95% CI: 52.4–82.5) and 60.3% (95% CI: 45.4–80.1) (Fig. 1a), respectively. The median LC was 87 months (95% CI: 50.4-not reached). The 4-year LC rate was 56.4% for tumors in the retroperitoneum and pelvis, and 91.7% for tumors in the extremities and trunk. Eighteen patients died. Among these, 11 patients died due to distant metastasis, 4 succumbed to local progression, 2 deaths were attributed to unknown causes, and 1 patient died from another disease (Myelodysplastic Syndrome, MDS). The median DMFS, PFS and OS were 54.7 (95% CI: 24.6-not reached), 24.3 (95% CI: 16.0–54.7) and 67.3 (95% CI: 39.8-not reached) months, respectively. The 4-year DMFS, PFS and OS were 50.3% (95% CI: 36.5–69.3), 36.7% (95% CI: 24.7–54.5) and 59.7% (95% CI: 45.7–77.9), respectively. The 5-year DMFS, PFS and OS were 44.0% (95% CI: 29.1–66.5), 28.0% (95% CI: 16.1–48.4) and 54.7% (95% CI: 39.9–75.0), respectively (Fig. 1b-d). Detailed survival estimates at 2, 3, 4, and 5 years for LC, PFS, DMFS, and OS are provided in Supplementary Table S3. Supplementary Table S4 summarizes the yearly event counts and censoring for local failure, distant metastasis, and death in patients with dedifferentiated liposarcoma (*n* = 8) and leiomyosarcoma (*n* = 5).

**Fig. 2:**
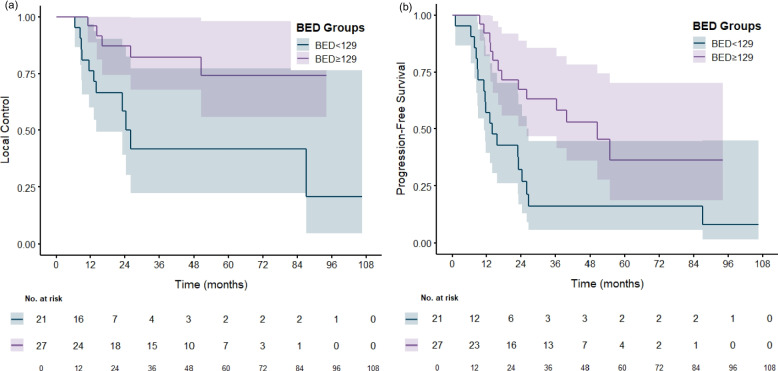
Kaplan–Meier analysis of local control (**a**) and progression-free survival (**b**) stratified by biologically effective dose (BED) in patients undergoing carbon ion radiotherapy

**Fig. 3:**
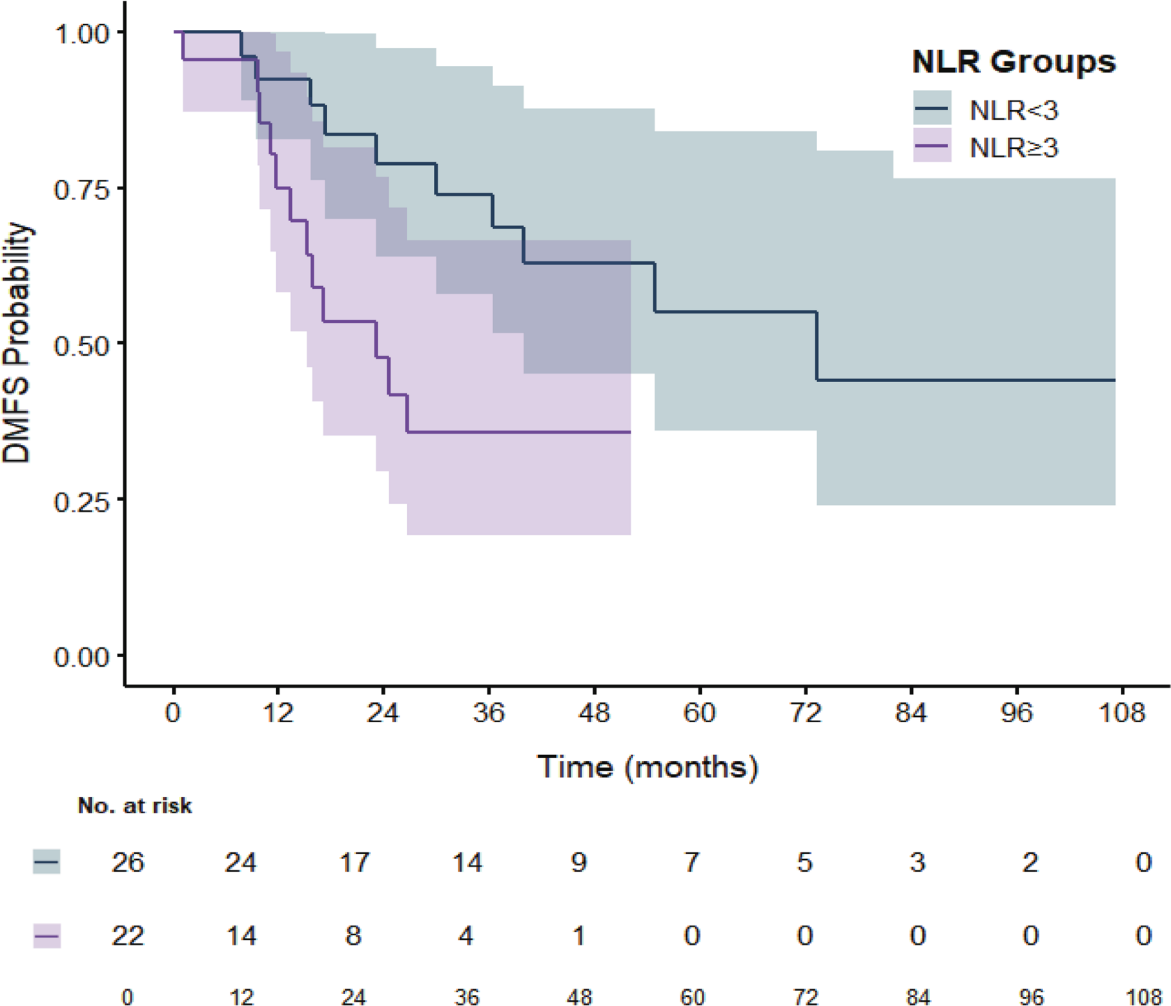
Kaplan–Meier analysis of distant metastasis free survival stratified by neutrophil-to-lymphocyte ratio in patients undergoing carbon ion radiotherapy

The results of the univariate analysis for factors associated with LC, PFS, and DMFS are presented in Table 2. Radiation dose demonstrated a significant impact on both LC and PFS. Patients who were administered a higher prescribed dose (BED ≥ 129 Gy, α/β = 5) demonstrated significantly superior LC (*p* = 0.010) and PFS (*p* = 0.004) compared with those who received a lower prescribed dose (Fig. 2). The 5-year LC was 41.7% (95% CI: 22.5%−77.2%) for patients in the BED < 129 Gy group, compared with 74.0% (95% CI: 55.8%−98.2%) for those in the BED ≥ 129 Gy group. The mean tumor volume was 148.6 cc (range: 5.5–2957.2 cc). A tumor size less than 150 cc was correlated with better PFS (*p* = 0.014), although it was not significantly associated with better LC (*p* = 0.322). The median survival for patients with tumor size less than 150 cc and those with tumors 150 cc or greater was 50.4 months and 22.8 months, respectively. The mean NLR was 3.56 ± 2.70 before treatment and 4.19 ± 3.71 after CIRT (*p* = 0.964). The median pre‑CIRT NLR was 2.79 (IQR, 2.0–4.13). The pre-CIRT NLR of 3 or higher was associated with worse DMFS (*p* = 0.020) (Fig. 3), whereas a post-CIRT NLR of 3 or higher was not significantly correlated with DMFS (*p* = 0.568). Other parameters were not significantly correlated with clinical outcomes (see Table 2).

**Table 2:** Univariate analysis

Variables	LC		DMFS		PFS		OS	
	HR (95% CI)	*P* value	HR (95% CI)	*P* value	HR (95% CI)	*P* value	HR (95% CI)	*P* value
Age (< 55y vs. ≥ 55y)	1.648 (0.615–4.417)	0.320	1.010 (0.432–2.364)	0.982	0.963 (0.474–1.959)	0.918	0.675 (0.266–1.713)	0.408
Tumor location (retroperitoneum and pelvis vs. trunk and extremities)	2.298 (0.786–6.724)	0.129	1.905 (0.769–4.716)	0.164	1.701 (0.791–3.658)	0.174	1.837 (0.662–5.101)	0.243
Previous radiotherapy (yes vs. no)	0.548 (0.124–2.414)	0.427	0.781 (0.207–2.942)	0.715	1.007 (0.348–2.915)	0.990	4.729 (0.946–23.65)	0.059
Metal implants (yes vs. no)	0.539 (0.124–2.346)	0.410	1.263 (0.336–4.749)	0.729	0.790 (0.267–2.341)	0.671	0.549 (0.130–2.331)	0.416
Radiation dose (BED < 129 Gy vs BED ≥ 129 Gy)	3.937 (1.398–11.090)	0.010*	1.884 (0.782–4.540)	0.158	3.067 (1.437–6.545)	0.004*	2.492 (0.962–6.459)	0.060
Tumor volume (GTV < 150 cc vs GTV ≥ 150 cc)	0.598 (0.217–1.652)	0.322	0.538 (0.230–1.258)	0.153	0.399 (0.192–0.829)	0.014*	0.503 (0.199–1.272)	0.146
NLR before treatment (NLR < 3 vs NLR ≥ 3)	0.925 (0.341–2.507)	0.878	0.333 (0.132–0.842)	0.020*	0.556 (0.267–1.157)	0.116	0.688 (0.260–1.822)	0.452
NLR after treatment (NLR < 3 vs NLR ≥ 3)	1.008 (0.363–2.794)	0.988	0.885 (0.379–2.065)	0.778	0.724 (0.357–1.466)	0.375	0.725 (0.286–1.836)	0.498

No acute Grade ≥ 2 hematologic, gastrointestinal, or systemic toxicities were recorded. The most frequently observed acute toxicity was radiation dermatitis. Grade 2 and grade 3 acute radiation dermatitis were observed in two patients each (4.2%), and all patients completed the planned radiotherapy course. Late toxicities included grade 2 dermatitis in six patients (12.5%) and grade 3 dermatitis in two patients (4.2%), primarily localized to the limb and vulvar region, respectively. Additional late complications included one case of grade 2 and one case of grade 3 decreased joint range of motion. Late neurological toxicities were observed in three patients: two cases of grade 2 peripheral neuropathy (4.2%) and one case of grade 3 peripheral neuropathy (2.1%). This grade 3 peripheral neuropathy was observed in a patient undergoing CIRT followed by surgical resection. All grade ≥ 2 acute and late toxicities were exclusively observed in patients who received a BED ≥ 129 Gy.

**Fig. 4:**
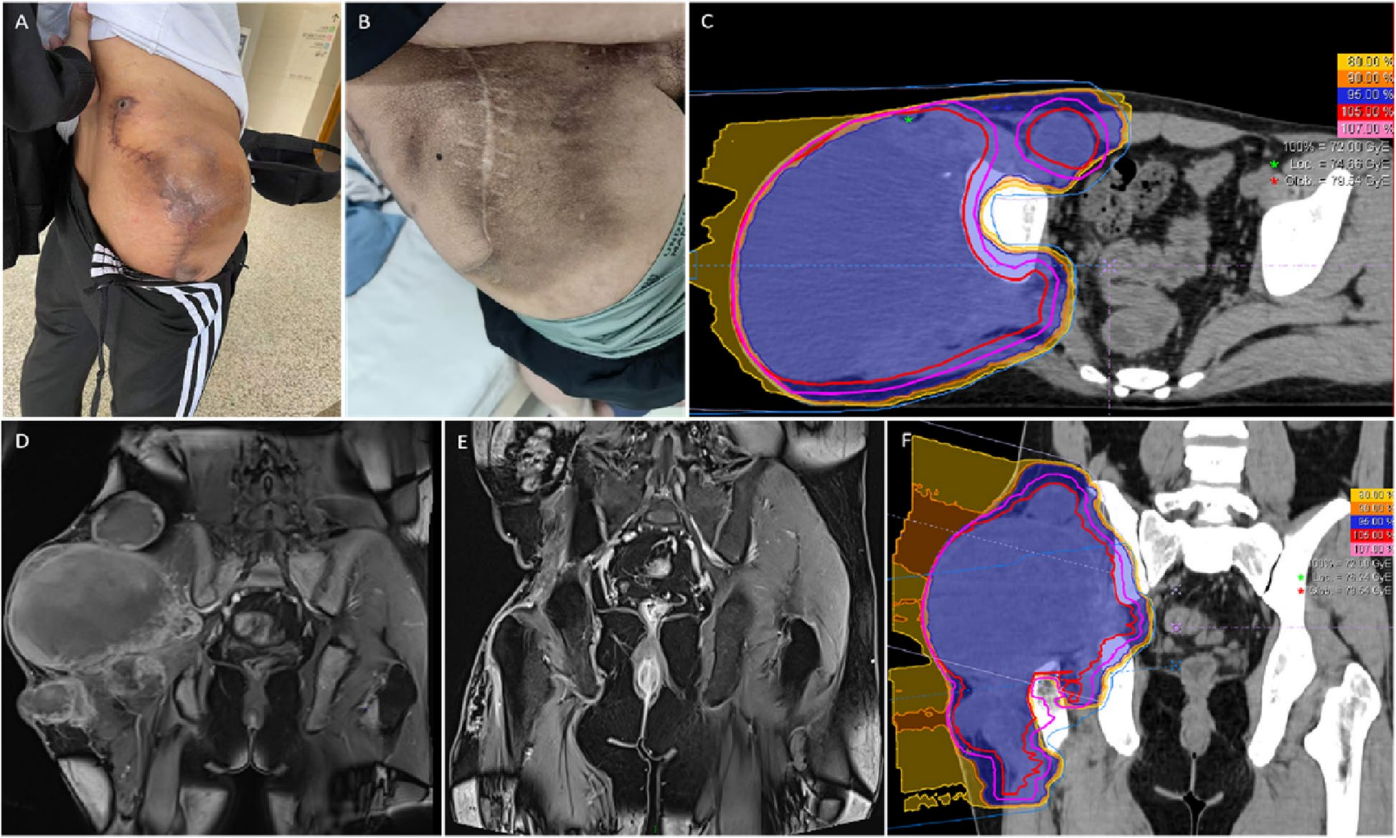
Management of a patient with recurrent CIC‑rearranged sarcoma. This patient presented with local recurrences two months after initial surgery. The patient received CIRT (72 Gy/18 fractions) followed by tumor resection one month later. **A**, **B** Clinical photographs of the skin at the lesion site before (**A**) and 3 years after (**B**) completion of CIRT and surgery. **C**, **F** Axial (**C**) and coronal (**F**) views of the CIRT dose distribution. The CTV was prescribed to receive ≥ 95% of the prescribed dose. **D**, **E** MRI before (**D**) and 3 years after (**E**) CIRT and surgery. In brief. For a detailed clinical summary, treatment parameters, and longitudinal outcome data of this case, please refer to Supplementary Case Report 1

Notably, following multidisciplinary team (MDT) review, three patients with tumors initially deemed unresectable due to large size and proximity to critical organs received neoadjuvant CIRT with radical intent doses, aiming for tumor downstaging to enable subsequent surgery. This strategy differed from definitive CIRT alone and was pursued based on MDT consensus that surgical resection might become feasible after significant tumor response. One month post-CIRT, tumor resection was performed in all three cases. The first case involved a patient with a Capicua transcriptional repressor (CIC)—rearranged sarcoma that recurred two months postoperatively, presenting with multiple tumors, the largest of which measured 15 × 17 cm. The patient received CIRT (72 Gy/18 fractions) followed by tumor resection one month later. At the latest follow-up (42.7 months), the patient remained disease-free. However, grade 3 late peripheral neuropathy presented as foot numbness and pain, ultimately requiring distal amputation followed by prosthetic rehabilitation. The patient currently exhibits normal ambulation and functional activity (Fig. 4). To preserve limb function, the second patient underwent preoperative CIRT with a total dose of 72 Gy delivered in 18 fractions. One month post-CIRT, the patient underwent complete surgical resection of the tumor. At the most recent follow-up (32.3 months), the patient remained disease-free with preserved limb function. The third case involved a patient with recurrent pelvic low-grade fibromyxoid sarcoma who received CIRT (70.4 Gy/16 fractions). Tumor resection was performed two months post-CIRT. Unfortunately, local recurrence developed at 11.1 months after surgery, followed by lung metastasis at 14.2 months after surgery. Metal implants within the radiation field were observed in this patient, potentially associated with tumor recurrence.

## Discussion

Surgical resection remains the standard curative-intent treatment for localized STS. However, population-based studies indicate that 15–55% of patients are considered ineligible for surgery due to anatomical limitations (e.g., tumor proximity to neurovascular bundles) or medical comorbidities [12]. Moreover, the management of locally recurrent disease presents significant therapeutic dilemmas, as these patients often face prohibitive reoperation risks or experience disease recurrence even after surgery.

Previous studies have demonstrated promising LC rates with CIRT for STS, such as the 5-year LC rate of 65% reported by Imai et al. [5]. In a study reported by Serizawa et al., among 24 patients with unresectable RPS received CIRT, the 5-year LC and OS rates were 69% and 50%, respectively [13]. More recently, a larger single-institution study of 76 patients with unresectable RPS treated with CIRT reported 3- and 5-year LC rates of 79.0% and 72.0%, and OS rates of 68.3% and 49.4%, further supporting its efficacy [14]. These results were superior to previous outcomes from photon therapy. However, these studies were primarily based on the MKM, which is predominantly used in Japanese facilities. In contrast, European centers and our institution utilize the LEM, which may yield different clinical outcomes [5, 6]. This study aims to fill this knowledge gap by evaluating the efficacy and safety of CIRT based on the LEM model.

Our study included 48 patients with unresectable primary or recurrent STS who were treated with CIRT at SPHIC. The median follow-up was 48.6 months, and the overall results demonstrated favorable LC and survival outcomes. The 4-year and 5-year LC rates were 65.8% (95% CI: 52.4%−82.5%) and 60.3% (95% CI: 45.4%−80.1%), respectively, indicating that CIRT can provide durable LC and consistent with those reported by Imai et al. [5]. Furthermore, the 4-year and 5-year OS rates were 59.7% and 54.7%, respectively. The median survival time was 67.3 months (95% CI: 39.8 to not reached). Previous study [15] from the Massachusetts General Hospital (MGH) included 112 patients underwent photon therapy and reported the 5-year LC, DFS, and OS were 45%, 24%, and 35%, respectively. Allignet et al. [12] report that, in 116 unresectable STS patients treated with definitive photon therapy, 3-year local failure (LF), PFS and OS were 43.2%, 16.6% and 34%, respectively. Median OS was 21.4 months. Our results are quite higher than those results. Systematic review also [16] showed that definitive photon therapy achieves 5-year LC rates of 28–73% in unresectable STS, while particle therapies achieve rates of 52–72%.

Supplementary Table S1 illustrates the substantial divergence in RBE-weighted doses between LEM and MKM models across our fractionation schemes. For example, our predominant 72 Gy/18 fraction regimen (LEM) corresponds to 63 Gy/18 fraction under MKM—a discrepancy that likely contributes to outcome differences between European/LEM-based and Japanese/MKM-based CIRT series.

All toxicities of grade ≥ 2 occurred in patients who received a BED ≥ 129 Gy. This observed toxicity profile was favorable and comparable to outcomes reported in MKM-based series [17], and experiences from European LEM-based centers [18], both of which similarly emphasize the elevated risk of neurotoxicity with high doses to the sacral nerves. Specifically, the risk substantially increases when over 10 cm of the nerve length is exposed to doses exceeding 70 Gy (RBE), as prescribed by the Japanese RBE model [17]. These findings suggest that while higher doses are associated with better LC, they may also increase the risk of toxicity. Therefore, careful dose optimization and individualized treatment planning are essential to balance efficacy and safety.

Several factors have been identified as potential predictors of clinical outcomes. Higher prescribed radiation doses (BED ≥ 129 Gy) were significantly associated with improved LC and PFS (LC: *p* = 0.010; PFS: *p* = 0.004). This finding highlights the importance of dose escalation in CIRT, which may be particularly critical for radio-resistant STS. While dose–response relationships are well established in photon radiotherapy [15], comparable evidence for CIRT remains limited and inconsistent [5]. This discrepancy likely originate from fundamental differences in radiobiological modeling approaches, where the conversion factor increases with decreasing prescribed dose. In this study, the predominant dose fractionation regimen in our cohort was 72 Gy delivered in 18 fractions (BED = 129.6 Gy, α/β = 5). Notably, Japanese protocols typically employ 70.4 Gy in 16 fractions (BED = 132.4 Gy, α/β = 5). It should be noted that Japan typically delivers four fractions per week, whereas our center delivers five fractions per week, though both approaches maintain comparable therapeutic ratios. Tumor volume emerged as an independent prognostic factor, with lesions < 150 cc demonstrating superior PFS outcomes (*p* = 0.014). This suggests that larger tumor volumes may pose a greater challenge for effective LC, potentially due to increased biological complexity or anatomical constraints near organs at risk.

To the best of our knowledge, this is the first study to evaluate the association between NLR and prognosis in STS patients who underwent CIRT. An elevated pre-CIRT NLR (≥ 3) predicted worse DMFS (*p* = 0.020), indicating that tumor-associated inflammation may influence the metastatic potential of STS. Several studies have investigated the prognostic value of NLR in various cancer types, including STS. A meta-analysis by Liu et al. confirmed that an elevated pretreatment NLR correlates with worse survival in STS patients [19]. Studies have also identified the NLR as an independent prognostic factor for OS in RPS and synovial sarcoma [20, 21]. Barcellini et al. [22], showed that ACC patients with higher pre-CIRT levels of NLR had a predictable decrease in DFS. However, the post-CIRT NLR lost its prognostic value (*p* = 0.568), our research findings are inconsistent with those of Martinez et al. [23], who used hypofractionated radiotherapy (30 Gy in 5 fractions) and showed that higher post-RT NLR and a value ≥ 4 was associated with worse DFS and DMFS. Therefore, the relationship between post-CIRT NLR and therapeutic efficacy after CIRT remains unclear, which may be related to the radiation field coverage of lymphocytes during CIRT. Preclinical evidence indicates carbon ions reduce myeloid-derived suppressor cells (MDSCs) and enhance antigen presentation, potentially resetting the immune contexture [24, 25]. Additionally, the neutrophil‑to‑lymphocyte ratio can be affected by potential confounders such as concurrent infection or corticosteroid use, which were not systematically captured in this analysis.

To address the potential confounding effect of prior chemotherapy on the NLR, an analysis was performed after excluding the four patients who received chemotherapy within one month prior to CIRT. As demonstrated in Supplementary Figure S1, the association between a pre-CIRT NLR ≥ 3 and worse distant metastasis-free survival (DMFS) remained statistically significant (*p* = 0.007) in the cohort of 44 patients. These findings support the robustness of the pre‑CIRT NLR as a prognostic marker, reflecting a baseline inflammatory state shaped by both tumor biology and prior treatment history, rather than merely acute chemotherapy‑induced changes.

Three patients in our study underwent multidisciplinary evaluation and received neoadjuvant CIRT followed by surgical resection due to the large tumor size and proximity to critical organs. Our study is the first, to our knowledge, to report the effect of neoadjuvant CIRT in unresectable STS. Two patients achieved disease-free survival at latest follow-up, highlighting the potential role of CIRT in a multimodal treatment strategy for tumors deemed unresectable or recurrent after prior surgery. Notably, all three patients received preoperative radiotherapy with a radical dose, rather than the conventional prophylactic radiation dose. One patient developed grade 3 late neurological toxicity, potentially associated with extensive tumor encasement of the nerve, as both CIRT and surgical intervention may induce neurotoxicity. The dose distribution and dose-volume histogram for this patient are illustrated in Supplementary Figure S2.

Recent advances in preoperative photon radiotherapy provide important context for interpreting these findings. An ultra-hypofractionated regimen (30 Gy/5 fractions over 5 days) demonstrated promising 2-year local control (92.4%) in a nonrandomized clinical trial of extremity/trunk STS, albeit with 30% major wound complications [26]. A moderately hypofractionated approach (42.75 Gy/15 fractions over 3 weeks) achieved comparable 4-year local control (93%) with remarkably low late toxicity (9%) [27, 28]. However, both trials strictly excluded retroperitoneal/pelvic tumors, recurrent disease, and lesions abutting critical neural structures—precisely the population comprising 75% and 83.3% of our cohort, respectively.

From a dosimetric perspective, the BED ≥ 129 Gy (α/β = 5) employed in this study substantially exceeds that of the aforementioned photon hypofractionated regimens. Although direct comparisons warrant caution, the 5-year local control rate achieved in this study (60.3%) was comparable to that reported by Imai et al. using MKM-based CIRT (65%) and superior to historical photon therapy outcomes from Massachusetts General Hospital (45%). These findings suggest that for complex anatomical sites where photon hypofractionation is technically constrained, the superior dose gradient characteristics of CIRT may offset the prolonged treatment duration, offering a unique dose-escalation pathway for unresectable or recurrent STS. Further studies are needed to explore the optimal sequencing and integration of CIRT with surgery and other modalities.

Despite providing promising results into the efficacy and safety of CIRT for unresectable primary or recurrent STS, this study has several limitations. First, as a single-center retrospective analysis, the study design may inherently introduce selection bias and confounding factors. More importantly, the patient cohort is relatively small (n = 48) and inherently heterogeneous, comprising both primary (16.7%) and recurrent (83.3%) cases, as well as patients with prior in-field radiotherapy (18.8%). These heterogeneities introduce potential biases that cannot be fully adjusted for given the limited sample size, and they may affect the generalizability of our findings. Second, the limited number of clinical events reduced statistical power. Given the commonly recommended minimum of 10 events per variable, multivariable Cox or logistic regression would be statistically underpowered and potentially misleading in our cohort. Therefore, we deliberately restricted our analyses to univariate models to avoid overfitting and spurious associations. The results should be interpreted as exploratory and hypothesis-generating rather than confirmatory. Third, the absence of a direct comparator group hinders conclusive comparisons regarding the relative advantages of CIRT in terms of LC and toxicity profiles. Future prospective, multicenter studies with larger cohorts and integrated biomarker analyses are essential to validate these findings, optimize patient stratification, and refine therapeutic strategies.

## Conclusions

In conclusion, our study demonstrates that CIRT based on the LEM model is an effective and relatively safe treatment option for patients with unresectable primary or recurrent STS. Higher radiation doses and smaller tumor volumes were associated with better clinical outcomes, while systemic inflammation (as indicated by NLR) may influence metastatic potential. Given the rarity of unresectable STS and the limited long‑term data on LEM‑based CIRT, the present findings should be interpreted as hypothesis-generating. Future research should focus on optimizing treatment protocols, exploring the role of CIRT in multimodal strategies, and validating these findings in larger cohorts. Additionally, future work should include a prospective randomized trial or a propensity‑score‑matched retrospective comparison with contemporary photon IMRT cohorts to allow for a more rigorous and direct evaluation of treatment efficacy and toxicity.

## Supplementary Information

Supplementary Table S1.

Supplementary Table S2.

Supplementary Table S3.

Supplementary Table S4.

Supplementary Figure S1. Kaplan–Meier analysis of distant metastasis-free survival according to baseline neutrophil-to-lymphocyte ratio in the analysis cohort (*n* = 44), which excluded patients receiving chemotherapy within one month prior to radiotherapy.

Supplementary Figure S2. Treatment planning and dose‑volume histogram (DVH) for the patient with CIC‑rearranged sarcoma. (a) Axial view of the carbon ion radiotherapy plan showing dose distribution; the clinical target volume (CTV) is outlined in pink, and the prescribed dose of 72 Gy (RBE) is delivered in 18 fractions. (b) DVH demonstrating the dose to the sacral nerve (highlighted in yellow).

Supplementary Case report S1.

## Data Availability

The datasets used and/or analysed during the current study available from the corresponding author on reasonable request.

## Abbreviations

CIRT: Carbon ion radiotherapy
STS: Soft tissue sarcoma
LC: Local control
OS: Overall survival
PFS: Progression-free survival
DMFS: Distant metastasis-free survival
LEM: Local Effect Model
MKM: Microdosimetric Kinetic Model
RBE: Relative biological effectiveness
BED: Biologically effective dose
NLR: Neutrophil-to-lymphocyte ratio
GTV: Gross tumor volume
CTV: Clinical target volume
MDT: Multidisciplinary team
