# Supplementary material for: Carbon ion radiotherapy for unresectable primary or recurrent soft tissue sarcomas: long-term outcomes based on the local effect model
Source: BMC Cancer. 2026 Jun 4;26:959. doi: 10.1186/s12885-026-16213-w (PMC13455471; doi:10.1186/s12885-026-16213-w)
Supplement: Supplementary file 1 [file 12885_2026_16213_MOESM5_ESM.pdf]

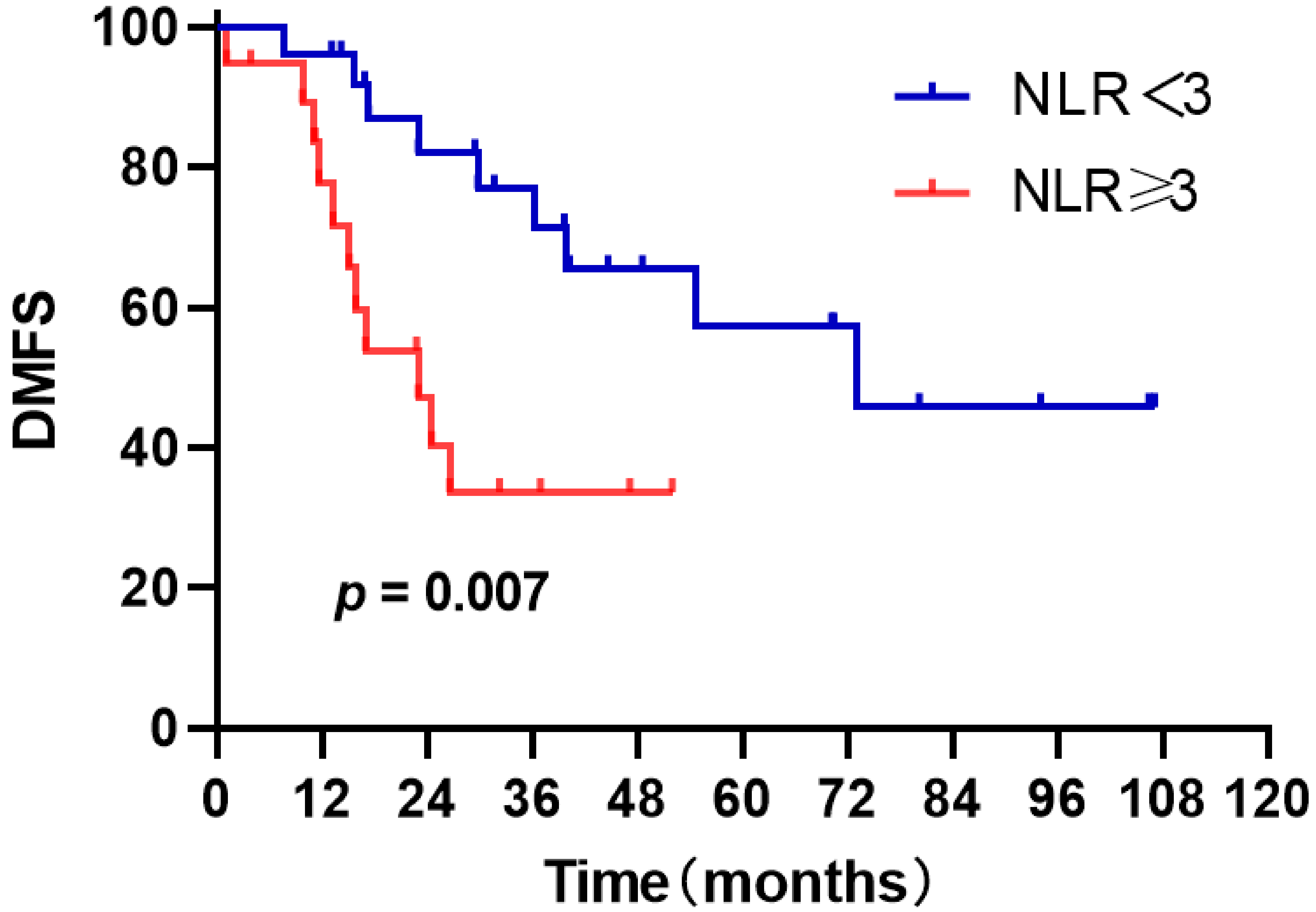

Number of risk

NLR<3	25	25	18	15	10	8	6	4	3	1
NLR≥3	19	14	8	4	2	0	0	0	0	0
