# Supplementary material for: Carbon ion radiotherapy for unresectable primary or recurrent soft tissue sarcomas: long-term outcomes based on the local effect model
Source: BMC Cancer. 2026 Jun 4;26:959. doi: 10.1186/s12885-026-16213-w (PMC13455471; doi:10.1186/s12885-026-16213-w)
Supplement: Supplementary file 2 [file 12885_2026_16213_MOESM6_ESM.pdf]

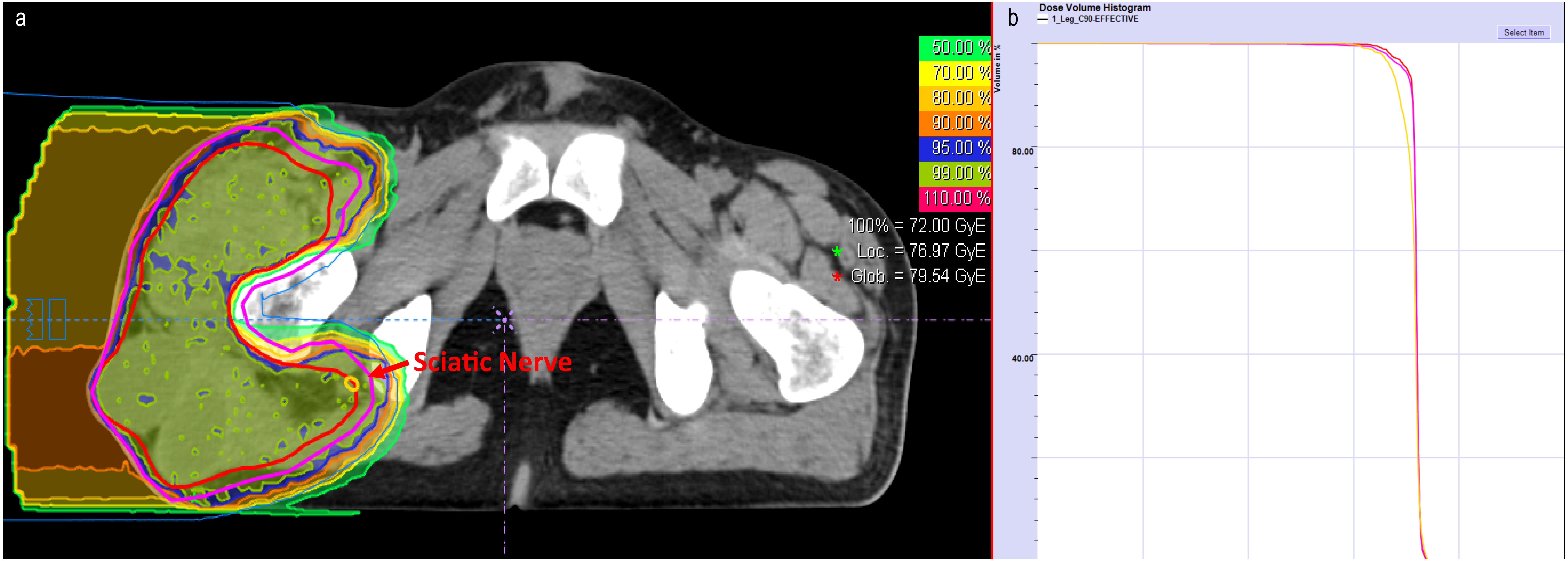
a
50.00 %
70.00 %
80.00 %
90.00 %
95.00 %
99.00 %
110.00 %
100% = 72.00 GyE
Loc. = 76.97 GyE
Glob. = 79.54 GyE
Sciatic Nerve
b
Dose Volume Histogram
1_Leg_C90-EFFECTIVE
Select Item
Volume in %
80.00
40.00
