# Supplementary material for: Carbon ion radiotherapy for unresectable primary or recurrent soft tissue sarcomas: long-term outcomes based on the local effect model
Source: BMC Cancer. 2026 Jun 4;26:959. doi: 10.1186/s12885-026-16213-w (PMC13455471; doi:10.1186/s12885-026-16213-w)
Supplement: Supplementary file 3 [file 12885_2026_16213_MOESM1_ESM.docx]

Supplementary Table S1. Fractionations of IMCT treatment scheme and comparison of LEM vs. MKM RBE dose

| No. of patients | Total dose  Gy(RBE) | Fractions | Dose per fraction | BED (α/β= 5) | MKM dose per fraction | MKM total dose |
| --- | --- | --- | --- | --- | --- | --- |
| 1 | 54 | 18 | 3 | 86.4 | 2.21 | 39.78 |
| 3 | 66 | 22 | 3 | 105.6 | 2.21 | 48.62 |
| 7 | 69 | 23 | 3 | 110.4 | 2.21 | 50.83 |
| 1 | 72 | 22 | 3.27 | 119.1 | 2.54 | 55.88 |
| 1 | 66 | 20 | 3.3 | 109.6 | 2.57 | 51.4 |
| 2 | 75.9 | 23 | 3.3 | 126 | 2.57 | 59.11 |
| 1 | 77.9 | 23 | 3.39 | 130.6 | 2.69 | 61.87 |
| 1 | 63 | 18 | 3.5 | 107.1 | 2.83 | 50.94 |
| 2 | 61.2 | 17 | 3.6 | 105.3 | 2.96 | 50.32 |
| 1 | 80.8 | 22 | 3.67 | 140.1 | 3.05 | 67.1 |
| 1 | 64.6 | 17 | 3.8 | 113.7 | 3.23 | 54.91 |
| 2 | 60 | 15 | 4 | 108 | 3.5 | 52.5 |
| 13 | 72 | 18 | 4 | 129.6 | 3.5 | 63 |
| 7 | 70.4 | 16 | 4.4 | 132.4 | 4.07 | 65.12 |
| 1 | 79.2 | 18 | 4.4 | 148.9 | 4.07 | 73.26 |
| 1 | 67.5 | 15 | 4.5 | 128.2 | 4.22 | 63.3 |
| 3 | 69 | 15 | 4.6 | 132.5 | 4.37 | 65.55 |
