# Supplementary material for: Carbon ion radiotherapy for unresectable primary or recurrent soft tissue sarcomas: long-term outcomes based on the local effect model
Source: BMC Cancer. 2026 Jun 4;26:959. doi: 10.1186/s12885-026-16213-w (PMC13455471; doi:10.1186/s12885-026-16213-w)
Supplement: Supplementary file 4 [file 12885_2026_16213_MOESM2_ESM.docx]

| **Histology** | **No. of patients** |
| --- | --- |
| Dedifferentiated liposarcoma | 8 |
| well‑differentiated liposarcoma | 1 |
| Myxoid liposarcoma | 1 |
| Leiomyosarcoma | 5 |
| Synovial sarcoma | 4 |
| Undifferentiated pleomorphic sarcoma (UPS) | 4 |
| Malignant peripheral nerve sheath tumor (MPNST) | 3 |
| Myxofibrosarcoma | 3 |
| Clear cell sarcoma | 2 |
| Rhabdomyosarcoma | 2 |
| Undifferentiated sarcoma | 2 |
| Mesenchymal malignancy | 2 |
| Malignant solitary fibrous tumor | 2 |
| Solitary fibrous tumor | 1 |
| CIC‑rearranged sarcoma | 1 |
| Spindle cell sarcoma | 1 |
| Post‑radiation sarcoma | 1 |
| Malignant Paraganglioma | 1 |
| Aggressive fibromatosis (desmoid tumor) | 1 |
| Fibroblastic sarcoma | 1 |
| Low-grade fibromyxoid sarcoma | 1 |
| Angiosarcoma | 1 |

Supplementary Table S2. Pathological Subtypes of 48 Soft Tissue Sarcoma Cases
