# Supplementary material for: Carbon ion radiotherapy for unresectable primary or recurrent soft tissue sarcomas: long-term outcomes based on the local effect model
Source: BMC Cancer. 2026 Jun 4;26:959. doi: 10.1186/s12885-026-16213-w (PMC13455471; doi:10.1186/s12885-026-16213-w)
Supplement: Supplementary file 5 [file 12885_2026_16213_MOESM3_ESM.docx]

Supplementary Table S3. Two- to five-year rates of local control (LC), progression-free survival (PFS), distant metastasis-free survival (DMFS), and overall survival (OS) in 48 patients with unresectable sarcoma. Data are presented as percentages with 95% confidence intervals.

|  | LC | PFS | DMFS | OS |
| --- | --- | --- | --- | --- |
| 2 years | 74.8% (95%CI 62.8-89.0) | 51.5% (95%CI 38.8-68.4) | 65.1% (95%CI 52.2-81.3) | 77.5% (95%CI 66.1-90.9) |
| 3 years | 65.8% (95%CI 52.4-82.5) | 42.1% (95%CI 27.6-56.0) | 57.1% (95%CI 43.7-74.6) | 69.4% (95%CI 56.7-85.0) |
| 4 years | 65.8% (95%CI 52.4-82.5) | 36.7% (95%CI 24.7-54.5) | 50.3% (95%CI 36.5-69.3) | 59.7% (95%CI 45.7-77.9) |
| 5 years | 60.3% (95%CI 45.4-80.1) | 28.0% (95%CI 16.1-48.4) | 44.0% (95%CI 29.1-66.5) | 54.7% (95%CI 39.9-75.0) |
