# Supplementary material for: Carbon ion radiotherapy for unresectable primary or recurrent soft tissue sarcomas: long-term outcomes based on the local effect model
Source: BMC Cancer. 2026 Jun 4;26:959. doi: 10.1186/s12885-026-16213-w (PMC13455471; doi:10.1186/s12885-026-16213-w)
Supplement: Supplementary file 6 [file 12885_2026_16213_MOESM4_ESM.docx]

Supplementary Table S4. Descriptive event counts and censoring at yearly intervals for dedifferentiated liposarcoma (n=8) and leiomyosarcoma (n=5)

(A) Dedifferentiated liposarcoma (n=8)

| Time interval (years) | Local failure (n) | | | | Distant metastasis (n) | | | | Death (n) | | | |
| --- | --- | --- | --- | --- | --- | --- | --- | --- | --- | --- | --- | --- |
|  | At risk | Event | Censored | At risk | | Event | Censored | At risk | | Event | Censored |  |
| 0-1 | 8 | 0 | 0 | 8 | | 1 | 0 | 8 | | 0 | 0 |  |
| 1-2 | 8 | 2 | 3 | 7 | | 2 | 1 | 8 | | 2 | 1 |  |
| 2-3 | 3 | 1 | 0 | 4 | | 1 | 0 | 5 | | 1 | 0 |  |
| 3-4 | 2 | 0 | 0 | 3 | | 1 | 1 | 4 | | 1 | 0 |  |
| 4-5 | 2 | 0 | 1 | 1 | | 0 | 0 | 3 | | 1 | 0 |  |
| ＞5 | 1 | 0 | 1 | 1 | | 0 | 1 | 2 | | 1 | 1 |  |

(B) Leiomyosarcoma (n=5)

| Time interval (years) | Local failure (n) | | | | Distant metastasis (n) | | | | Death (n) | | | |
| --- | --- | --- | --- | --- | --- | --- | --- | --- | --- | --- | --- | --- |
|  | At risk | Event | Censored | At risk | | Event | Censored | At risk | | Event | Censored |  |
| 0-1 | 5 | 1 | 0 | 5 | | 2 | 0 | 5 | | 0 | 0 |  |
| 1-2 | 4 | 0 | 1 | 3 | | 0 | 0 | 5 | | 0 | 1 |  |
| 2-3 | 3 | 0 | 0 | 3 | | 0 | 0 | 4 | | 0 | 0 |  |
| 3-4 | 3 | 0 | 1 | 3 | | 1 | 0 | 4 | | 1 | 0 |  |
| 4-5 | 2 | 0 | 1 | 2 | | 1 | 1 | 3 | | 0 | 2 |  |
| ＞5 | 1 | 0 | 1 | 0 | | 0 | 0 | 1 | | 0 | 1 |  |

Notes:

- For each endpoint, the risk set (“At risk”) is defined independently as the number of patients who have not yet experienced the endpoint event and have not been censored prior to the start of the interval.
- Censored: patients who were lost to follow-up or alive without the event of interest at the end of the interval. For each endpoint, censoring was defined independently.
- Due to the small sample size, these data are descriptive only and formal statistical comparisons are not performed.
