# Supplementary material for: Carbon ion radiotherapy for unresectable primary or recurrent soft tissue sarcomas: long-term outcomes based on the local effect model
Source: BMC Cancer. 2026 Jun 4;26:959. doi: 10.1186/s12885-026-16213-w (PMC13455471; doi:10.1186/s12885-026-16213-w)
Supplement: Supplementary file 7 [file 12885_2026_16213_MOESM7_ESM.docx]

**Case Report for figure 4: Long-term Control with Grade 3 Neuropathy in STS Treated with Neoadjuvant CIRT and Surgery**

**Patient:** 29-year-old male
**Diagnosis:** Capicua transcriptional repressor (CIC) - rearranged sarcoma

**Location:** Pelvic

**Clinical Timeline & Management**

**1. Initial Presentation**

- 15×17×13 cm mass, deep to fascia, adjacent to sciatic nerve
- MRI: Huge mass in the right pelvic (Figure 4D-E)
- Core biopsy: Capicua transcriptional repressor (CIC) - rearranged sarcoma
- Treatment history: The tumor recurred two months after radical resection.

**2. Neoadjuvant CIRT**

- IMCT, D_RBE_: 72 Gy / 18 fractions
- Target: GTV + 0.5-1.0 cm margin
- Acute toxicity: Grade 3 late peripheral neuropathy

**3. Surgical Resection**

- Time: One month after CIRT
- Wide local excision
- Margins: R0
- Pathology: Most of the tumor necrosis

**4. Postoperative Course**

- **1-2 months after resection:** Initial wound healing, mild neuropathy
- **9 months after resection:** Grade 2 neuropathy (foot numbness)
- **24 months after resection:** Due to foot numbness and worsening limited mobility, the patient underwent a lower limb amputation (Grade 3 neuropathy).
- **30 months after resection:**  Prosthetic fitting, functional rehabilitation
- **Until now:** He is able to walk, drive, and perform daily activities normally

**Outcomes Summary**

- **Local control:** 54 months (no recurrence at last follow-up)
- **Complication:** Grade 3 sciatic neuropathy → amputation at 24 months after tumor resection
- **Functional status:** Independent ambulation with prosthesis

**Clinical Significance**

This case illustrates:

1. Durable local control achievable with neoadjuvant CIRT
2. Grade 3 sciatic neuropathy potentially associated with extensive tumor encasement of the nerve, as both CIRT and surgical intervention may induce neurotoxicity.
3. Importance of long-term functional assessment in limb-salvage protocols
